# Supplementary material for: Minimal Infiltrative Disease Identification in Cryopreserved Ovarian Tissue of Girls with Cancer for Future Use: A Systematic Review
Source: Cancers (Basel). 2023 Aug 22;15(17):4199. doi: 10.3390/cancers15174199 (PMC10486797; doi:10.3390/cancers15174199)
Supplement: Supplementary file 1 [file cancers-15-04199-s001.zip › Grubliauskaite et al_Table S1.pdf]

Table S1. GRADE criteria adapted for the systematic review for detection of minimally infiltrative disease in cryopreserved pediatric ovarian tissue. RCT = Randomised Controlled Trial.

<table>
<tr><th>GRADE criteria</th><th>Rating</th><th>Reasons for
down- or upgrading</th><th>Quality of the evidence</th></tr>
<tr><td>Study design</td><td>RCT (High)
Non-RCT (Low)</td><td>Only non-RCTs</td><td rowspan="7">High
⊕⊕⊕⊕

Moderate
⊕⊕⊕⊖

Low
⊕⊕⊖⊖

Very low
⊕⊖⊖⊖</td></tr>
<tr><td>Study limitations</td><td>No
Minimal (-1)
Important (-2)</td><td>Consideration of covariates including:
• Selection bias
• Attrition bias
• Measurement bias
• Detection bias
• Study confounding
• Statistical analysis</td></tr>
<tr><td>Consistency</td><td>No
Serious (-1)</td><td>Lack of replication
Similar studies showing contradicting results</td></tr>
<tr><td>Directness</td><td>No
Serious (-1)</td><td>Generalisability</td></tr>
<tr><td>Precision</td><td>No
Serious (-1)
Very serious (-2)</td><td>Small sample sizes
One study included</td></tr>
<tr><td>Publication Bias</td><td>Unlikely
Strongly suspected (-1)</td><td>Not all data presented without explanation</td></tr>
<tr><td>Other (upgrading factors)</td><td>Effect size (+1 or +2)
Dose-response (+1 or +2)
Plausible confounding (+1 or +2)</td><td></td></tr>
</table>
