# Supplementary material for: Minimal Infiltrative Disease Identification in Cryopreserved Ovarian Tissue of Girls with Cancer for Future Use: A Systematic Review
Source: Cancers (Basel). 2023 Aug 22;15(17):4199. doi: 10.3390/cancers15174199 (PMC10486797; doi:10.3390/cancers15174199)
Supplement: Supplementary file 1 [file cancers-15-04199-s001.zip › Grubliauskiate et al_Table S2.pdf]

Table S2: Evidence tables of 17 articles included in the systemic review.

Question		
**[15]** ***Schifflers et al.*** Microscopic infiltration of cryopreserved ovarian tissue in 2 patients with Ewing sarcoma. Journal of Pediatric Hematology/Oncology. 2018; 40(3): e167-e170		
**Study design**<br>**Treatment period**<br>**Years of follow-up**	**Participants**	**Markers and methods used**
Study design: Case reports<br><br>Study period: 2001-2012<br><br>Follow-up:<br>• No follow-up.	Type and number of non-participants: n=0<br><br>Type and number of participants: pediatric, n=2<br><br>Diagnoses: Ewing sarcoma<br><br>Treatment before OTC: No<br><br>Age at OTC: 6-13<br><br>Controls: primary tumor markers<br><br>Additional study characteristics/ confounders:<br>• Both patients in remission after 11 months post completion of therapy.	Markers studied:<br>• CD99<br>• *EWSR1* rearrangements<br><br>Methods used:<br>• immunohistochemistry (CD99)<br>• FISH (*EWSR1* rearrangement)
**1. markers and techniques**		
**Main outcomes**<br>**markers**	**Additional remarks**	
Outcome definitions: Presence of malignant cells defined if the expression of CD99 or *EWRS1* rearrangements were found.<br><br>Results: though no distant metastasis were found in the other parts of the body, in OT of both patients, infiltration of malignant cells was confirmed by IHC and FISH.<br><br>Risk factors/ determinants:<br>• initial workup did not detect malignant cell infiltration in ovarian tissues of both patients.	Strengths:<br>• two methods used to confirm the presence of EWS cells;<br>• primary tumor control.<br><br>Limitations:<br>• initial workup did not detect malignant cell infiltration in ovarian tissues of both patients.<br><br>**Risk of bias**<br>A. Selection bias:<br>high risk<br>Reason: two participants<br><br>B. Attrition bias:<br>low risk<br>Reason: both participants gave outcomes: presence/absence of markers	C. Measurement bias:<br>low risk<br>Reason: valid and reliable method used to detect the markers<br><br>D. Detection bias:<br>low risk<br>Reason: valid and reliable method used to detect presence or absence of minimal infiltrative disease<br><br>E. Confounding:<br>low risk<br>Reason: all confounders (treatment before OTC, availability of original tumor/blood markers, survival status/disease recurrence) are accounted<br><br>F. Statistical analysis and reporting:<br>low risk<br>Reason: all data presented

Question
[16] ***Diaz-Garcia et al.*** Dexamethasone does not prevent malignant cell reintroduction in leukemia patients undergoing ovarian transplant: risk assessment of leukemic cell transmission by a xenograft model, Human Reproduction, 2019; 34(8):1485–93

**Study design**<br>**Treatment period**<br>**Years of follow-up**	**Participants**	**Markers and methods used**
Study design: retrospective study<br><br>Study period: before 2019<br><br>Follow-up:<br>• 2 patients deceased and for the rest 3 no follow-up.	Type and number of non-participants: n=1, age not specified<br><br>Type and number of participants: pediatric, n=5<br><br>Diagnoses: ALL<br><br>Treatment before OTC: all patients had treatment prior OTC<br><br>Age at OTC: 3-15<br><br>Controls: markers of primary tumor<br><br>Additional study characteristics:<br>• in 1 patient MID was already found before OTC;<br>• tissue pieces were 5x5x1mm.	Markers studied:<br>• *IgH* rearrangements<br>• *EA2-PBX1*<br><br>Methods used:<br>• reverse-transcription droplet digital PCR (*IgH* rearrangements, *EA2-PBX1*)<br>• histology<br>• xenotransplantation

**1. markers and techniques**		
**Main outcomes**<br>**markers**	**Additional remarks**	
Outcome definitions: Presence of malignant cells defined if the expression of *IgH* rearrangements or fusion transcript were discovered.<br><br>Results: The ovarian grafts were negative for malignancy on macroscopic and pathological evaluation. After xenotransplantation and RT-PCR analysis 4 patients were found positive with markers, though out of 22 fragments grafted only 7 were positive which means not every fragment contains malignant cells.<br><br>Risk factors/ determinants:<br>• not all primary tumors have specific markers;<br>• all patients received chemotherapy before OTC;<br>• patient that had MID detected before OTC was deceased.	Strengths:<br>• multiple fragments of one patient xenografted and evaluated;<br>• after xenotransplantation other organs of mice were tested for metastatic cells;<br>• primary tumor markers.<br><br>Limitations:<br>• 1 patient not screen for PCR due to absence of molecular markers.<br><br>**Risk of bias**<br>A. Selection bias:<br>high risk<br>Reason: only basic characteristics of patients provided, low sample size<br><br>B. Attrition bias:<br>low risk<br>Reason: all provided some kind of outcome: presence/absence of markers	C. Measurement bias:<br>low risk<br>Reason: valid and reliable method to detect the markers<br><br>D. Detection bias:<br>moderate risk<br>Reason: valid and reliable methods used to detect presence or absence of MID except for one patient<br><br>E. Confounding:<br>low risk<br>Reason: all confounders (treatment before OTC, availability of original tumor/blood markers, survival status/disease recurrence) are accounted<br><br>F. Statistical analysis and reporting:<br>low risk<br>Reason: all data presented

Question		
**[24]** ***Abir et al.*** Occasional involvement of the ovary in Ewing sarcoma. Human reproduction 2010; 25(7): 1708-1712		
**Study design**<br>**Treatment period**<br>**Years of follow-up**	**Participants**	**Markers and methods used**
Study design: retrospective cohort study<br><br>Study period: 2000-2009<br><br>Follow-up:<br>• follow-up and autotransplantation was done for patient 7.	Type and number of non-participants: adult, n=1<br><br>Type and number of participants: pediatric, n=7<br><br>Diagnoses: Ewing sarcoma (EWS)<br><br>Treatment before OTC: patient 1 after 6 cycles of ADR, VP16, I-FOS, ACTD, C, VCR + thoracic radiation; patient 4 after VCAIE and patient 6 after 2 courses of VCR, D, C, E, I-FOS<br><br>Age at OTC: 13-17<br><br>• Controls: primary tumors (though data not shown) and peripheral blood (PB) samples of 2,4,7 patients were studied for RT-PCR.<br><br>Additional study characteristics/confounders:<br>• 4 patients had OTC before treatment;<br>• 1 patient died from malignancy (patient 2);<br>• each patient's thawed ovarian tissue fragment (2x2cm^2) (except nr6) were used for immunohistochemistry;<br>• RNA was extracted twice from two different areas of ovary for patients 2,4,5,6,7;<br>• in case of patient 5, positive PCR of OT obtained only from first round.	Markers studied:<br>• CD99<br>• *EWS-FLI 1* fusion transcript<br><br>Methods used:<br>• immunohistochemistry (IHC) (CD99)<br>• reverse-transcription PCR and nested PCR (*EWS-FLI 1* fusion transcript)<br>• histology
**1. markers and techniques**		
**Main outcomes**<br>**markers**	**Additional remarks**	
Outcome definitions: Presence of malignant cells defined if the expression of the fusion transcript or CD99 were discovered.<br><br>Results: The ovarian specimens of 1-5, 7, 8 were negative for malignancy on pathological evaluation. IHC did not yield evidence of malignancy. In 1 out of 5 ovaries RT-PCR showed presence of *EWS-FLI 1* fusion transcript. In 1 out of 3 PB samples RT-PCR showed presence of *EWS-FLI 1* fusion transcript (not the same patient as detected in ovarian sample).<br><br>Risk factors/ determinants:<br>• no CD99 or *EWS-FLI 1* fusion transcripts found in patients who had treatment before OTC – treatment could have had influence.	Strengths:<br>• peripheral blood tested for 3 patients next to ovarian tissue RT-PCR and immunohistochemistry;<br>• pathologists did the evaluation of immunohistochemistry;<br>• two samples from different sites of ovary were tested for some patients by RT-PCR;<br>• positive primary tumor samples positive for CD99.<br><br>Limitations:<br>• peripheral blood tested only for 3 patients;<br>• not all ovarian tissue samples investigated by IHC and RT-PCR.<br><br>**Risk of bias**<br>A. Selection bias:<br>high risk	C. Measurement bias:<br>moderate risk<br>Reason: not all patients had their ovarian tissue analyzed for markers by RT-PCR<br><br>D. Detection bias:<br>moderate risk<br>Reason: not all patients had their ovarian tissue analyzed by the same methods to detect presence or absence of minimal infiltrative disease<br><br>E. Confounding:<br>low risk

	Reason: low sample size<br><br><u>B. Attrition bias:</u><br>moderate risk<br>Reason: not all participants gave an outcome: presence/absence of markers	Reason: all confounders (treatment before OTC, availability of original tumor/blood markers, survival status/disease recurrence) are accounted<br><br><u>F. Statistical analysis and reporting:</u><br>moderate risk<br>Reason: unclear why some ovarian tissues were not analyzed the same way; could be selective reporting

Question		
**[25]** ***Chaput et al.*** Sensitive and specific detection of Ewing sarcoma minimal residual disease in ovarian and testicular tissues in an in vitro model. Cancers 2019; 11: 1807		
**Study design**<br>**Treatment period**<br>**Years of follow-up**	**Participants**	**Markers and methods used**
Study design: Clinical trial/prospective cohort study<br><br>Study period: 2017<br><br>Follow-up:<br>• No follow-up.	Type and number of non-participants: boys, n=3<br><br>Type and number of participants: girls, n=5<br><br>Diagnoses: Ewing sarcoma (EWS)<br><br>Treatment before OTC: chemotherapy<br><br>Age at OTC: 13-16<br><br>Controls: primary tumor markers<br><br>Additional study characteristics/confounders:<br>• 2 deceased and 3 alive patients;<br>• two out of 3 alive patients had metastasis.	Markers studied:<br>• *EWS-FLI 1* fusion transcript<br>• 5' and 3' sites of *EWSR1* gene<br><br>Methods used:<br>• reverse-transcription qPCR (*EWS-FLI 1* fusion transcript)<br>• FISH<br>• histology
**1. markers and techniques**		
**Main outcomes**<br>**markers**	**Additional remarks**	
Outcome definitions: Presence of malignant cells defined if the expression of the fusion transcript or *EWSR1* were discovered.<br><br>• Results The ovarian specimens were negative for malignancy on pathological evaluation. RT-qPCR and FISH showed absence of minimal infiltrative disease (MID) (*EWS-FLI 1* fusion transcript and *EWSR1* accordingly).<br><br>Risk factors/ determinants:<br>• chemotherapy before OTC.	Strengths:<br>• sensitive molecular methods used for detection – RT-qPCR and FISH;<br>• primary tumors for specific patient markers used;<br>• patients with metastasis evaluated.<br><br>Limitations:<br>• deceased patients evaluated;<br>• no xenotransplantation done.<br><br>**Risk of bias**<br>A. Selection bias:<br>high risk<br>Reason: only baseline characteristics of patients provided and low sample size<br><br>B. Attrition bias:<br>low risk<br>Reason: all patients gave an outcome: presence/absence of markers	C. Measurement bias:<br>low risk<br>Reason: valid and reliable methods used to detect markers<br><br>D. Detection bias:<br>low risk<br>Reason: valid and reliable methods used to detect presence or absence of MID<br><br>E. Confounding:<br>low risk<br>Reason: all confounders (treatment before OTC, availability of original tumor/blood markers, survival status/disease recurrence) are accounted<br><br>F. Statistical analysis and reporting:<br>low risk<br>Reason: all data presented

Question		
**[26]** ***Yding Andersen et al.*** No malignancy detected in surplus ovarian tissue from a former Ewing sarcoma patient who experienced relapse four years after being grafted with frozen/thawed ovarian tissue. Journal of Assisted Reproduction and Genetics 2014; 31: 1567-1568		
**Study design**<br>**Treatment period**<br>**Years of follow-up**	**Participants**	**Markers and methods used**
Study design: Letter to the editor about a case report<br><br>Study period: 2005<br><br>Follow-up:<br>• autotransplantation was done with 2 ovarian tissue fragments. After 4.5 years of OT transplantation patient relapsed and died after 7 months.	Type and number of non-participants: n=0<br><br>Type and number of participants: pediatric, n=1<br><br>Diagnoses: Ewing sarcoma (EWS)<br><br>Treatment before OTC: No<br><br>Age at OTC: 9<br><br>Controls: primary tumor markers<br><br>Additional study characteristics/confounders:<br>• all remaining frozen tissue pieces evaluated.	Markers studied:<br>• *EWS/FLI* translocation<br><br>Methods used:<br>• PCR (*EWS/FLI*)
**1. markers and techniques**		
**Main outcomes**<br>**markers**	**Additional remarks**	
Outcome definitions: Presence of malignant cells defined if the expression of translocation found.<br><br>Results: All ovarian specimens were negative for malignancy by PCR.<br><br>Risk factors/ determinants:<br>• N/A	Strengths:<br>• all remaining tissue pieces evaluated;<br>• primary tumor control.<br><br>Limitations:<br>• Original transplanted OT could not be tested for malignancies.<br><br>**Risk of bias**<br>A. Selection bias:<br>high risk<br>Reason: one participant<br><br>B. Attrition bias:<br>low risk<br>Reason: participant gave an outcome: presence/absence of marker	C. Measurement bias:<br>low risk<br>Reason: valid and reliable method used to detect the marker<br><br>D. Detection bias:<br>low risk<br>Reason: valid and reliable method used to detect presence or absence of MID<br><br>E. Confounding:<br>low risk<br>Reason: all confounders (treatment before OTC, availability of original tumor/blood markers, survival status/disease recurrence) are accounted<br><br>F. Statistical analysis and reporting:<br>low risk<br>Reason: all data presented

Question		
**[27]** ***Greve et al.*** Ovarian tissue cryopreserved for fertility preservation from patients with Ewing or other sarcomas appear to have no tumor cell contamination. European Journal of Cancer 2013; 49: 1932-1938		
**Study design**<br>**Treatment period**<br>**Years of follow-up**	**Participants**	**Markers and methods used**
Study design: retrospective study<br><br>Study period: before 2013<br><br>Follow-up:<br>• no follow-up	Type and number of non-participants: adults, n=7<br><br>Type and number of participants: pediatric, n=9<br><br>Diagnoses: Ewing, synovial and osteosarcomas (OST)<br><br>Treatment before OTC: 1 patient had 7 cycles of VAI and VID; 2 patients had 1 cycle of VIDE, 1 patient had cisplatin, doxorubicin and methotrexate, 5 patients did not have treatment<br><br>Age at OTC: 7-16<br><br>Controls: primary tumor markers (only Ewing sarcoma patients)<br><br>Additional study characteristics:<br>• 1 patient had metastatic disease and 1 recurrence at the time of OTC;<br>• 5 x 5 x 1 mm size of OT, which was 3-11% (depending on a patient) of transplanted ovary.	Markers studied:<br>• *EWS-FLI 1* fusion gene<br><br>Methods used:<br>• reverse-transcription qPCR (*EWS-FLI 1* fusion gene)<br>• histology<br>• xenotransplantation
**1. markers and techniques**		
**Main outcomes**<br>**markers**	**Additional remarks**	
Outcome definitions: Presence of malignant cells defined if the expression of *EWS-FLI 1* fusion gene was found (for EWS patients) or after xenotransplantation if mice or their organs showed presence of malignant cells by histology (for all patients).<br><br>Results: The ovarian specimens before and after xenotransplantation were negative for malignancy on pathological evaluation. In all EWS patients RT-qPCR showed absence of MID in OT.<br><br>Risk factors/ determinants:<br>• chemotherapy before OTC.	Strengths:<br>• OT evaluated before and after xenotransplantation;<br>• primary tumor evaluated for markers with RT-PCR or FISH.<br><br>Limitations:<br>• only Ewing sarcoma patients evaluated by RT-qPCR;<br>• *EWS-FLI1* was not found in one primary tumor sample of EWS patient.<br><br>**Risk of bias**<br>A. Selection bias:<br>high risk<br>Reason: low sample size<br><br>B. Attrition bias:<br>low risk<br>Reason: all participants gave some kind of outcome: presence/absence of markers	C. Measurement bias:<br>moderate risk<br>Reason: not all patients had their ovarian tissue analyzed for markers with valid and reliable methods<br><br>D. Detection bias:<br>moderate risk<br>Reason: not all patients had their ovarian tissue analyzed for presence or absence of MID the same way<br><br>E. Confounding:<br>low risk<br>Reason: all confounders (treatment before OTC, availability of original tumor/blood markers, survival status/disease recurrence) are accounted<br><br>F. Statistical analysis and reporting:<br>moderate risk

		Reason: study is more valuable for EWS than OTS or synovial sarcoma patients

Question		
**[28]** ***Hoekman et al.*** Searching for metastasis in ovarian tissue before autotransplantation: a tailor-made approach. Fertility and sterility 2015; 103(2): 469-477		
**Study design**<br>**Treatment period**<br>**Years of follow-up**	**Participants**	**Markers and methods used**
Study design: retrospective studies<br><br>Study period: 2002-2012<br><br>Follow-up:<br>• No follow-up.	Type and number of non-participants: adult, n=38-45<br><br>Type and number of participants: pediatric, n=2-9 (EWS: 1-3 and OTS: 1-6)<br><br>Diagnoses: Ewing sarcoma (EWS), osteosarcoma (OST)<br><br>Treatment before OTC: 1 EWS patient had chemotherapy<br><br>Age at OTC: EWS mean 19y (range 17-21) and OTS mean 15y (range 13-18)<br><br>Controls: primary tumor markers<br><br>Additional study characteristics/confounders:<br>• 2 EWS patients and 1 OST deceased.	Markers studied:<br>• CD99<br>• *EWS-FLI 1* fusion transcript<br><br>Methods used:<br>• immunohistochemistry (IHC) (CD99);<br>• reverse-transcription PCR (*EWS-FLI 1* fusion transcript)<br>• histology
**1. markers and technique**		
**Main outcomes**<br>**markers**	**Additional remarks**	
Outcome definitions: Presence of malignant cells defined if the expression of fusion transcript or CD99 staining were discovered or histology fit the primary tumor histology.<br><br>Results: The ovarian specimens were negative for malignancy on pathological evaluation. IHC and RT-PCR confirmed the absence of minimal infiltrative disease (MID).<br><br>Risk factors/ determinants:<br>• One EWS patient received treatment before OTC.	Strengths:<br>• primary tumors for specific patient markers;<br>• IHC and RT-PCR used for detection of MID in EWS patients.<br><br>Limitations:<br>• Age presented in mean (range) from which we cannot say how many actually patients were children;<br>• Not all OTS or EWS patients' ovarian tissues analyzed, cannot be sure if analyzed ones were children;<br>• no xenotransplantation done;<br>• OST had no specific antibodies available – evaluated by histology.<br><br>**Risk of bias**<br>A. Selection bias:<br>high risk<br>Reason: no information of inclusion and exclusion criteria, low sample size<br><br>B. Attrition bias:<br>high risk<br>Reason: only a part of all participant were evaluated	C. Measurement bias:<br>high risk<br>Reason: valid and reliable method used to detect a marker for EWS patients; OTS only histology<br><br>D. Detection bias:<br>high risk<br>Reason: valid and reliable method used to detect a marker for EWS patients; OTS only histology<br><br>E. Confounding:<br>moderate risk<br>Reason: though all important ones are accounted, due to poor representation of patients it is impossible to understand for whom they are for<br><br>F. Statistical analysis and reporting:<br>high risk<br>Reason: not enough data to have adequate analysis or how many pediatric patients were evaluated

Question		
**[29]** ***Dolmans et al.*** Evaluation of minimal disseminated disease in cryopreserved ovarian tissue from bone and soft tissue sarcoma patients. Human reproduction 2016; 31(10): 2292-2302		
**Study design**<br>**Treatment period**<br>**Years of follow-up**	**Participants**	**Markers and methods used**
Study design: retrospective study<br><br>Study period: not mentioned, but before 2016<br><br>Follow-up:<br>• No follow-up.	Type and number of non-participants: girls/woman with no markers found in primary tumor tissue, n=21; Li-Fraumeni syndrome patient, n=1; adults n=10;<br><br>Type and number of participants: pediatric, n=16<br><br>Diagnoses: Ewing sarcoma (EWS), primitive neuroectodermal tumor (PNET) (Ewing family) and soft tissue sarcomas (STS): rhabdomyosarcoma, synovial sarcoma, clear cell sarcoma<br><br>Treatment before OTC: 2 rhabdomyosarcoma patients had treatment before OTC<br><br>Age at OTC: 1-17<br><br>Controls: original tumor markers, healthy human ovaries and fibrous scar tissue<br><br>Additional study characteristics/confounders:<br>• 3 EWS, 1 rhabdomyosarcoma (deceased) and 1 clear cell sarcoma patients had metastasis, 1 rhabdomyosarcoma (deceased) had recurrence and 2 PNET were deceased;<br>• amount of tissue used – less than 10% for each patient.	Markers studied:<br>• CD99<br>• Myogenin<br>• MyoD I<br>• Bcl-2<br>• NSE,<br>• S100,<br>• MDM2<br>• Melanoma cocktail<br>• *EWS-FLI 1, EWS-ETV1, EWSRI-ERG, PAX3-FOXO1, PAX7-FOXO1, MyoD1, SYT-SSX, EWS-ATF1* fusion transcripts<br><br>Methods used:<br>• histology<br>• immunohistochemistry (IHC) (CD99, myogenin, MyoD I, Bcl-2, NSE, S100, MDM2, melanoma cocktail)<br>• reverse-transcription PCR (*EWS-FLI 1, EWS-ETV1, EWSR1-ERG, PAX3-FOXO1, PAX7-FOXO1, MyoD1, SYT-SSX, EWS-ATF1*)<br>• FISH (only for primary tumors: *FLI 1/EWSR1, EWSR1/ERG, PAX3, PAX7, LSI EWSR1, LSI FKHR/FOXO1, LSI SYT/SS18*)
**1. markers and techniques**		
**Main outcomes**<br>**markers**	**Additional remarks**	
Outcome definitions: Presence of malignant cells defined if the expression of gene translocations or protein staining were discovered.<br><br>Results: The ovarian specimens were negative for malignancy on pathological evaluation. Moreover, IHC did not yield evidence of malignancy. RT-qPCR showed absence of evaluated gene translocations.<br><br>Risk factors/ determinants:<br>• 2 STS patients had undergone chemotherapy prior OTC;<br>• 2 STS patients were analyzed though had no primary tumor markers.	Strengths:<br>• IHC and RT-qPCR-used for molecular markers in ovarian tissue;<br>• primary tumors for specific patient markers detected by IHC and FISH;<br><br>Limitations:<br>• In 22 patients (including adults) no patient-specific markers were found in primary tumor tissue;<br>• Not all ovarian tissues were evaluated by PCR due to unavailability of original tumor tissue or no markers found in original tumor tissue;<br>• Only 4 out of 8 STS patients had their tissues analyzed by RT-qPCR;<br><br>**Risk of bias**<br>A. Selection bias:<br>high risk	C. Measurement bias:<br>moderate risk<br>Reason: not all patients had their ovarian tissue analyzed for markers by RT-qPCR<br><br>D. Detection bias:<br>moderate risk<br>Reason: not all patients had their ovarian tissue analyzed for markers by RT-qPCR<br><br>E. Confounding:<br>moderate risk<br>Reason: no information about treatment prior OTC for EWS patients<br><br>F. Statistical analysis and reporting:

	Reason: no information about inclusion criteria and recruitment and low sample size<br><br><u>B. Attrition bias:</u><br>low risk<br>Reason: all patients gave some kind of outcome: presence/absence of markers	low risk<br>Reason: all data presented

Question		
**[30]** ***Abir et al.*** Ovarian minimal residual disease in chronic myeloid leukaemia. Reproductive BioMedicine Online 2014; 24: 255-260		
**Study design**<br>**Treatment period**<br>**Years of follow-up**	**Participants**	**Markers and methods used**
Study design: Case report<br><br>Study period: 1999<br><br>Follow-up: follow-up of at least 12 years	Type and number of non-participants: none<br><br>Type and number of participants: pediatric, n=1<br><br>Diagnoses: chronic myeloid leukamia (CML)<br><br>Treatment before OTC: No<br><br>Age at OTC: 12 years<br><br>Controls: markers in bone marrow and peripheral blood at the time of diagnosis<br><br>Additional study characteristics/ confounders:<br>• no recurrence of the disease for 12 years	Markers studied:<br>• myeloperoxidase<br>• glycophorin A<br>• CD34<br>• CD68<br>• LCA/DC45<br>• Factor VIII<br>• *BCR-ABL* transcript<br><br>Methods used:<br>• immunohistochemistry (IHC) (myeloperoxidase, glycophorin A, CD34, CD68, LCA/DC45, Factor VIII)<br>• quantitative reverse-transcription PCR and two step nested PCR (*BCR-ABL* transcript)<br>• histology
**1. markers and techniques**		
**Main outcomes**<br>**markers**	**Additional remarks**	
Outcome definitions: Presence defined by *BCR-ABL/ABL* transcripts ratio was 0.05% and PCR products were present on agarose gel. Autotransplantation was not recommended.<br><br>Results: The ovarian specimens were negative for malignancy on pathological evaluation. IHC did not yield evidence of malignancy. Frequency of leukemia-initiating cells by qPCR in the ovarian tissue of the patient was extremely low (0,05%). Two step nested PCR showed *BCR-ABL* transcript.<br><br>Risk factors/ determinants:<br>• Very low frequency of the transcript.	Strengths:<br>• many different markers studied and methods used to evaluate presence of malignant cells;<br>• peripheral blood tested;<br>• pathologists did the evaluation of IHC.<br><br>Limitations:<br>• extremely low frequency of qPCR products;<br>• no xenotransplantation done;<br>• contradiction in discussion – they say in results that in peripheral blood found no *BCR-AML* transcripts but the low frequency in ovarian tissue can be due to peripheral blood in the tissue.<br><br>**Risk of bias**<br>A. Selection bias:<br>high risk<br>Reason: one patient participation	B. Attrition bias:<br>Low risk<br>Reason: participant gave an outcome: presence/absence of markers<br><br>C. Measurement bias:<br>low risk<br>Reason: valid and reliable methods used to detect markers<br><br>D. Detection bias:<br>low risk<br>Reason: valid and reliable methods used to detect presence or absence of minimal infiltrative disease<br><br>E. Confounding:<br>low risk<br>Reason: all confounders (treatment before OTC, availability of original tumor/blood markers, survival status/disease recurrence) are accounted<br><br>F. Statistical analysis and reporting:<br>low risk<br>Reason: all data presented

Question		
**[31]** ***Soares et al.*** Eliminating malignant cells from cryopreserved ovarian tissue is possible in leukaemia patients. British Journal of Haematology 2017; 178: 231-239		
**Study design**<br>**Treatment period**<br>**Years of follow-up**	**Participants**	**Markers and methods used**
Study design: retrospective study<br><br>Study period: 1999-2011<br><br>Follow-up:<br>• No follow-up	Type and number of non-participants: adults, n=4<br><br>Type and number of participants: pediatric, n=8<br><br>Diagnoses: acute lymphoblastic leukemia (ALL), acute myeloid leukemia (AML)<br><br>Treatment before OTC: 1 round of cortisone and cyclophosphamide for 3 patients; 5 patients did not have treatment before OTC<br><br>Age at OTC: 4-17<br><br>Controls: markers from blood or bone marrow at the time of diagnosis<br><br>Additional study characteristics/confounders:<br>• for patient 4 leukemic cells were detected at the time of OTC;<br>• 8 out of 12 patients were deceased, not specified which ones; study cohort is not representative for alive patients;<br>• patients 3 and 11 had no molecular markers at the time of diagnosis, therefore did not have their OT evaluated by PCR.	Markers studied:<br>• *ETV6-RUNX1*<br>• *BCR-ABL1*<br>• *IgH*<br>• *TCR*<br>• *FLT3*<br><br>Methods used:<br>• RT-PCR (*ETV6-RUNX1, BCR-ABL1* fusion transcripts)<br>• PCR (*IGH, TCR, FLT3* genes)<br>• histology<br>• xenotransplantation
**1. markers and techniques**		
**Main outcomes**<br>**markers**	**Additional remarks**	
Outcome definitions: Presence of malignant cells defined if the expression of markers found.<br><br>Results: The ovarian specimens were negative for malignancy on pathological evaluation except for patient 4. PCR found ALL cells present in OT in 4 patients and AML cells present in 1 patient. After xenotransplantation only patients 2 and 3 had leukemic masses found in mice. Others did not though PCR was performed on the recovered tissues.<br><br>Risk factors/ determinants:<br>• 3 patients had chemotherapy started before OTC<br>• relation between lymphoblast blood count before OTC and positive result of PCR in ovarian tissue but had an exception with one patient – high count, no development of disease.	Strengths:<br>• checked for leukemic cells in OT, digested tissue suspension and follicle samples;<br>• pathologists did the evaluation of OT histology;<br>• different markers used for MID detection according to markers found in BM.<br><br>Limitations:<br>• not all patients had the same nucleic acid evaluated: patients 6 and 9 had RNA, and the rest – DNA isolated for detection of fusion transcripts;<br>• not all patients have specific molecular markers found (patients 3 and 11), no PCR performed for them.<br><br>**Risk of bias**<br>A. Selection bias:<br>high risk<br>Reason: study cohort is not representative for alive patients, majority are deceased	B. Attrition bias:<br>low risk<br>Reason: all participants gave an outcome: presence/absence of markers<br><br>C. Measurement bias:<br>moderate risk<br>Reason: not all patients had their ovarian tissue analyzed for markers the same way<br><br>D. Detection bias:<br>moderate risk<br>Reason: not all patients had their ovarian tissue analyzed for presence or absence of MID the same way<br><br>E. Confounding:<br>moderate risk

		Reason: though all confounders (treatment before OTC, availability of original tumor/blood markers, survival status/disease recurrence) are accounted, not all of them are specified to which patient<br><br>F. Statistical analysis and reporting:<br>low risk<br>Reason: all data presented

Question		
**[32]** ***Rodriguez-Wallberg et al.*** Successful pregnancies after transplantation of ovarian tissue retrieved and cryopreserved at time of childhood acute lymphoblastic leukemia – a case report. Heamatologica 2021; 106(10): 2783-2787		
**Study design**<br>**Treatment period**<br>**Years of follow-up**	**Participants**	**Markers and methods used**
Study design: Case report<br><br>Study period: 2001<br><br>Follow-up<br>• autotransplantation was done in 2017 and 2018. Healthy pregnancy after IVF in 2019 and spontaneous pregnancy in 2021.	Type and number of non-participants: n=0<br><br>Type and number of participants: pediatric, n=1<br><br>Diagnoses: ALL<br><br>Treatment before OTC: NOPHO ALL 2000 high risk protocol<br><br>Age at OTC: 14<br><br>Controls: markers in blood<br><br>Additional study characteristics/confounders:<br>• 8 pieces of 1-2x4mm OT evaluated;<br>• No recurrence after at least of 5 years.	Markers studied:<br>• *BCR-ABL* fusion transcript<br><br>Methods used:<br>• PCR (*BCR-ABL*)
**1. markers and techniques**		
**Main outcomes**<br>**markers**	**Additional remarks**	
Outcome definitions: Presence of malignant cells defined if the expression of fusion transcript found.<br><br>Results: The ovarian specimens absent of *BCR-ABL* fusion transcript.<br><br>Risk factors/ determinants:<br>• Chemotherapy before OTC.	Strengths:<br>• multiple tissue pieces evaluated before autotransplantation;<br>• reliable method (PCR) used for evaluation;<br>• patient's blood used for detecting the fusion transcript.<br><br>Limitations:<br>• OTC done after chemotherapy.<br><br>**Risk of bias**<br>A. Selection bias:<br>high risk<br>Reason: one participant<br><br>B. Attrition bias:<br>low risk<br>Reason: participant gave an outcome: presence/absence of marker	C. Measurement bias:<br>low risk<br>Reason: valid and reliable method used to detect the marker<br><br>D. Detection bias:<br>low risk<br>Reason: valid and reliable method used to detect presence or absence of MID<br><br>E. Confounding:<br>low risk<br>Reason: all confounders (treatment before OTC, availability of original tumor/blood markers, survival status/disease recurrence) are accounted<br><br>F. Statistical analysis and reporting:<br>low risk<br>Reason: all data presented

Question		
**[33]** ***Rosendahl et al.*** Evidence of residual disease in cryopreserved ovarian cortex from female patients with leukemia. Fertility and Sterility 2010; 94(6): 2186-2190		
**Study design**<br>**Treatment period**<br>**Years of follow-up**	**Participants**	**Markers and methods used**
Study design: retrospective study<br><br>Study period: before 2010<br><br>Follow-up:<br>• No follow-up.	Type and number of non-participants: n=12<br><br>Type and number of participants: pediatric, n=14<br><br>Diagnoses: ALL, CML, AML, JMML<br><br>Treatment before OTC: 18 participants in total had chemotherapy before OTC<br><br>Age at OTC: 2-17<br><br>Controls: markers in blood and bone marrow<br><br>Additional study characteristics/ confouders:<br>• OTC has been performed either during complete remission, chronic phase or active phase of disease;<br>• 1-2 pieces of ovarian tissue used (5-10 x 5-10 mm).	Markers studied:<br>• *TEL-AML1*<br>• *BCR-ABL b2a2* or *BCR-ABL b2a2/e1a2*<br>• *CBFB-MYH11* type A<br>• CD34<br>• CD10<br>• CD20<br>• CD79<br>• CD3<br>• TdT<br>• CD117<br>• MPO<br>• CD68<br><br>Methods used:<br>• immunohistochemistry (IHC) (CD34, CD10, CD20, CD79, CD3, TdT, CD117, MPO, CD68);<br>• PCR (*TEL-AML1, BCR-ABL b2a2 / b2a2/e1a1, CBFB-MYH11* type A)<br>• histology
**1. markers and techniques**		
**Main outcomes**<br>**markers**	**Additional remarks**	
Outcome definitions: Presence of malignant cells defined if the expression of markers found.<br><br>Results: The ovarian specimens were negative for IHC markers. 3 out of 5 ovarian tissue analyzed by PCR were positive for fusion transcript.<br><br>Risk factors/ determinants:<br>• Two pieces of OT examined by PCR but different results were observed for one patient;<br>• Chemotherapy before OTC.	Strengths:<br>• many biomarkers checked;<br>• two pieces of OT from each patient examined by PCR.<br><br>Limitations:<br>• Only 5 out of 14 patients examined by PCR;<br>• 9 patients' tumors had no specific markers;<br>• No xenotransplantation done (would be more reliable when no primary tumor markers or PCR give different result).<br><br>**Risk of bias**<br>A. Selection bias:<br>high risk<br>Reason: not adequately described and low sample size<br><br>B. Attrition bias:<br>low risk<br>Reason: all participants gave an outcome: presence/absence of markers	C. Measurement bias:<br>high risk<br>Reason: not all patients evaluated with valid and reliable method to detect markers<br><br>D. Detection bias:<br>high risk<br>Reason: only 5 of 14 evaluated OTs with valid and reliable method for MID<br><br>E. Confounding:<br>moderate risk<br>Reason: though all confounders (treatment before OTC, availability of original tumor/blood markers, survival status/disease recurrence) are accounted, not specified to which patient<br><br>F. Statistical analysis and reporting:<br>low risk<br>Reason: all data presented

Question		
**[34]** ***Zver et al.*** Minimal residual disease detection by multicolor flow cytometry in cryopreserved ovarian tissue from leukemia patients. Journal of Ovarian Research 2022; 15:9		
**Study design**<br>**Treatment period**<br>**Years of follow-up**	**Participants**	**Markers and methods used**
Study design: retrospective study<br><br>Study period: 2004-2018<br><br>Follow-up:<br>• No follow-up.	Type and number of non-participants: adults, n=7<br><br>Type and number of participants: pediatric, n=8<br><br>Diagnoses: B-ALL, T-ALL, AML<br><br>Treatment before OTC: all patients received chemotherapy before OTC<br><br>Age at OTC: 5-15<br><br>Controls: Leukemia-associated immunophenotypes (LAIP) and/or molecular markers of original tumor at the time of diagnosis<br><br>Additional study characteristics/ confounders:<br>• 1-2 mm^3 of OT used for MFC;<br>• no information on survival/disease recurrence status of the patients.	Markers studied:<br>• CD19, CD34, CD10-, negative for myeloid markers/ CD45, HLA-DR2, CD10, CD19, CD22, CD33/ CD19, CD10, CD22, CD38/ CD45, CD10, CD19, CD22, CD34, HLA-DR2/ CD2, cyCD3, CD5, CD7, CD10, CD33, CD34, CD45RA, CD123<br><br>Methods used:<br>• multicolor flow cytometry (MFC)<br>• xenotransplantation
**1. markers and techniques**		
**Main outcomes**<br>**markers**	**Additional remarks**	
Outcome definitions: Presence of malignant cells defined if the expression of LAIP were found.<br><br>Results: MFC detected positive level of minimal infiltrative disease (MID) in 2 patients, additionally, 1 positive event of LAIP had 3 patients each (considered negative result). After xenotransplantation, no MID found in bone marrow, blood, spleen and lymph nodes of mice. One mice died from weight loss which is considered to be a symptom of renewed disease (not confirmed by any other method).<br><br>Risk factors/ determinants:<br>• chemotherapy before OTC;<br>• for 2 patients after xenotransplantation both grafts not found and for 3 patients – one graft found.	Strengths:<br>• LAIP found at the diagnosis used for MID detection in OT;<br>• Xenotransplantation performed and other organs tested.<br><br>Limitations:<br>• due to limited amount of OT, for two patients xenotransplantation was not performed;<br>• due to limited amount of OT, RT-qPCR for molecular markers was not performed;<br>• OTC done in different institutions.<br><br>**Risk of bias**<br>A. Selection bias:<br>high risk<br>Reason: low sample size<br><br>B. Attrition bias:<br>low risk<br>Reason: all participants gave an outcome: presence/absence of markers	C. Measurement bias:<br>moderate risk<br>Reason: some of the patients did not have the same methods used for marker evaluation<br><br>D. Detection bias:<br>low risk<br>Reason: valid and reliable methods used to find presence or absence of MID<br><br>E. Confounding:<br>moderate risk<br>Reason: not all confounders (treatment before OTC, availability of original tumor/blood markers, survival status/disease recurrence) are accounted<br><br>F. Statistical analysis and reporting:<br>low risk<br>Reason: all data presented

Question		
**[35]** ***Asadi-Azarbaijani et al.*** Minimal residual disease of leukemia and the quality of cryopreserved ovarian tissue in vitro. Leukemia & lymphoma 2016; 57(3): 700-707		
**Study design**<br>**Treatment period**<br>**Years of follow-up**	**Participants**	**Markers and methods used**
Study design: retrospective study<br><br>Study period: before 2016<br><br>Follow-up:<br>• No follow-up.	Type and number of non-participants: adults, n=5<br><br>Type and number of participants: pediatric, n=9<br><br>Diagnoses: ALL, Burkitt's leukemia, AML<br><br>Treatment before OTC: yes, except one patient<br><br>Age at OTC: 1-16<br><br>Controls: original tumor markers<br><br>Additional study characteristics:<br>• tissue pieces of 2-3 x 3-4 x 1.5 mm^3 were used for each experiment; sensitivity of RT-qPCR ranged 0,0003-1%;<br>• all patients in remission.	Markers studied:<br>• *E2A-PBX1*<br>• *IgH*<br>• *TEL-AML1*<br>• *MLL-AF4*<br>• *AML1-ETO*<br>• *IgK Kde*<br>• *TCRD*<br>• *TCRB*<br><br>Methods used:<br>• RT-qPCR (*E2A-PBX1, IgH, TEL-AML1, MLL-AF4, AML1-ETO, IgK Kde, TCRD, TCRB*)<br>• histology
**1. markers and techniques**		
**Main outcomes**<br>**markers**	**Additional remarks**	
Outcome definitions: Presence of malignant cells defined if the expression of molecular markers were found.<br><br>Results: The ovarian specimens were negative for malignancy on histological evaluation. In OT of 2 patients, minimal infiltrative disease (MID) was detected and in 7 patients RT-qPCR showed absence of MID.<br><br>Risk factors/ determinants:<br>• different cryoprotectant agents used;<br>• all patients received chemotherapy before OTC;<br>• after cultivation *in vitro,* one patient (that was absent of MID in OT after thawing) showed positive result after 7 days.	Strengths:<br>• two markers checked if possible;<br>• high sensitivity for RT-qPCR;<br>• culture *in vitro* used.<br><br>Limitations:<br>• different fragments of OT can give different results (before and after *in vitro* culture).<br><br>**Risk of bias**<br>A. Selection bias:<br>high risk<br>Reason: low sample size<br><br>B. Attrition bias:<br>low risk<br>Reason: all participants gave an outcome: presence/absence of markers	C. Measurement bias:<br>low risk<br>Reason: valid and reliable method used to detect the markers<br><br>D. Detection bias:<br>low risk<br>Reason: valid and reliable method used to detect presence or absence of MID<br><br>E. Confounding:<br>low risk<br>Reason: all confounders (treatment before OTC, availability of original tumor/blood markers, survival status/disease recurrence) are accounted<br><br>F. Statistical analysis and reporting:<br>low risk<br>Reason: all data presented

Question		
**[36]** ***Dolmans et al.*** Reimplantation of cryopreserved ovarian tissue from patients with acute lymphoblastic leukemia is potentially unsafe. Blood 2010; 116(16): 2908-2914		
**Study design**<br>**Treatment period**<br>**Years of follow-up**	**Participants**	**Markers and methods used**
Study design: retrospective study<br><br>Study period: 1999-2008<br><br>Follow-up:<br>• No follow-up.	Type and number of non-participants: adult, n=8<br><br>Type and number of participants: pediatric, n=10<br><br>Diagnoses: CML, ALL<br><br>Treatment before OTC: 5 patients had one round of methotrexate and cortisone; 1 patient had one round of methotrexate and aspataginase, 4 had no treatment<br><br>Age at OTC: 3-17<br><br>Controls: cytogenetic abnormality or gene rearrangement present in blood or bone marrow<br><br>Additional study characteristics:<br>• less than 10% used of total cryopreserved OT for each patient;<br>• ovarian grafts presenting as tumor masses did not show follicular development.	Markers studied:<br>• *BCR-ABL1* fusion gene<br>• *Ig* and/or *TCR-gamma* rearrangement genes<br><br>Methods used:<br>• reverse-transcription qPCR (*BCR-ABL1*, *Ig* and/or *TCR-gamma* rearrangement genes)<br>• histology<br>• xenotransplantation
**1. markers and techniques**		
**Main outcomes**<br>**markers**	**Additional remarks**	
Outcome definitions: Presence of malignant cells defined if the expression of *BCR-ABL1* fusion gene or Ig and/or *TCR-gama* gene rearrangements were found or macro/microscopic evaluation of the grafts were infiltrated with leukemic cells.<br><br>Results: The ovarian specimens were negative for malignancy on pathological evaluation. In 5 patients RT-qPCR showed presence of molecular markers in OT before xenotransplantation. After xenotransplantation, leukemic cells were found in 4 grafts by microscopic evaluation, 2 out of them had metastasized to liver. Two grafts were positive for markers by RT-qPCR.<br><br>Risk factors/ determinants:<br>• 6 patients have already started chemotherapy before OTC;<br>• after xenotransplantation, grafts for 8 patients were not evaluated by RT-qPCR (insufficient RNA amount).–	Strengths:<br>• cytogenetic abnormality or gene rearrangement present in blood or bone marrow as controls;<br>• long-term xenotransplantation.<br><br>Limitations:<br>• 2 ALL patients did not show any molecular markers – no RT-qPCR done on their OT;<br>• only 2 patients evaluated by RT-qPCR after xenotransplantation.<br><br>**Risk of bias**<br>A. Selection bias:<br>high risk<br>Reason: low sample size<br><br>B. Attrition bias:<br>low risk<br>Reason: all participants gave an outcome: presence/absence of markers	C. Measurement bias:<br>moderate risk<br>Reason: not all patients had their ovarian tissue analyzed for markers with valid and reliable methods<br><br>D. Detection bias:<br>moderate risk<br>Reason: not all patients had their ovarian tissue analyzed for presence or absence of MID the same way<br><br>E. Confounding:<br>low risk<br>Reason: all confounders (treatment before OTC, availability of original tumor/blood markers, survival status/disease recurrence) are accounted<br><br>F. Statistical analysis and reporting:<br>low risk<br>Reason: all data presented

Question		
**[37]** ***Nguyen et al.*** Is ovarian tissue transplantation safe in patients with central nervous system primitive neuroectodermal tumors? Clinical Medicine 2020; 9: 4101		
**Study design**<br>**Treatment period**<br>**Years of follow-up**	**Participants**	**Markers and methods used**
Study design: case reports<br><br>Study period: 2001-2012<br><br>Follow-up:<br>• autotransplantation was done for patient 1 – three live births, relapse after 6 years post OT transplantation and death, patient 2 died and patient 3 is free of disease.	Type and number of non-participants: n=0<br><br>Type and number of participants: pediatric, n=3<br><br>Diagnoses: PNET<br><br>Treatment before OTC: No<br><br>Age at OTC: 4-17<br><br>Controls: primary tumor markers<br><br>Additional study characteristics/confounders:<br>• Patient 1 had metastasis at the diagnosis, others – localized disease.<br>• Two patients deceased, one is alive.	Markers studied:<br>• GFAP<br>• NSE<br>• ENO2<br><br>Methods used:<br>• Immunohistochemistry (IHC) (NSE, GFAP)<br>• reverse-transcription droplet digital PCR (RT-ddPCR) (*GFAP*, *ENO2*)<br>• histology<br>• xenotransplantation<br>• NGS
**1. markers and techniques**		
**Main outcomes**<br>**markers**	**Additional remarks**	
Outcome definitions: Presence of malignant cells were considered if concentration of the marker gene was equal or above limit of detection or IHC staining had a positive result.<br><br>Results: The ovarian specimens were negative for malignancy on pathological evaluation. IHC showed absence of NSE and GFAP in all patients OT. RT-ddPCR showed absence of *GFAP* in cryopreserved OT before or after xenotransplantation of 5 months. NGS for patient 1 did not show any specific mutations.<br><br>Risk factors/ determinants:<br>• *ENO2* is not specific marker for minimal infiltrative disease (MID).	Strengths:<br>• done all main three experimental methods for MID detection (IHC, RT-ddPCR, xenotransplantation);<br>• after xenotransplantation house-keeping gene transcript level stayed stable;<br>• NGS performed to find specific mutations for patient 1 in primary cancer;<br>• primary tumor control;<br>• analysis performed on relapsed/deceased and still alive patients.<br><br>Limitations:<br>• No specific mutations (markers) found for patient 1.<br><br>**Risk of bias**<br>A. Selection bias:<br>high risk<br>Reason: low sample size	B. Attrition bias:<br>low risk<br>Reason: all patients gave an outcome: presence/absence of markers<br><br>C. Measurement bias:<br>low risk<br>Reason: valid and reliable method used to detect markers<br><br>D. Detection bias:<br>low risk<br>Reason: valid and reliable method used to detect presence or absence of MID<br><br>E. Confounding:<br>low risk<br>Reason: all confounders (treatment before OTC, availability of original tumor/blood markers, survival status/disease recurrence) are accounted<br><br>F. Statistical analysis and reporting:<br>low risk<br>Reason: all data presented

<table>
<tr><th colspan="3">Question</th></tr>
<tr><td colspan="3">[38] Nguyen et al. Ovarian tissue cryopreservation and transplantation in patients with central nervous system tumours. Human reproduction 2021; 36(5): 1296-1309</td></tr>
<tr><th>Study design
Treatment period
Years of follow-up</th><th>Participants</th><th>Markers and methods used</th></tr>
<tr><td>Study design: prospective study

Study period: 2001-2018

Follow-up:
• no follow-up;
• one patient underwent OT transplantation.</td><td>Type and number of non-participants: adult, n=3

Type and number of participants: pediatric, n=17

Diagnoses: medulloblastoma, ependymoma, primitive neuroectodermal tumours (PNET), astrocytoma, glioblastoma, CNS germinoma

Treatment before OTC: No

Age at OTC: 13-17

Controls: primary tumor markers

Additional study characteristics/confounders:
• For 3 patients primary tumors were not available for RT-ddPCR;
• 1 PNET patient had metastasis and 5 had recurrence (not specified);
• Not specified which patients were alive or deceased;
• 9.1-16.7% of collected OT was used for MID and xenotransplantation.</td><td>Markers studied:
• NSE
• GFAP
• ENO2

Methods used:
• Immunohistochemistry (IHC) (NSE, GFAP)
• reverse-transcription droplet digital PCR (RT-ddPCR) (GFAP, ENO2)
• histology
• xenotransplantation</td></tr>
<tr><th colspan="3">1. markers and techniques</th></tr>
<tr><th>Main outcomes
markers</th><th>Additional remarks</th><th></th></tr>
<tr><td>Outcome definitions: Presence of malignant cells were considered if concentration of marker gene was equal or above limit of detection or IHC staining had a positive result.

Results: The ovarian specimens were negative for malignancy on pathological evaluation. Moreover, IHC did not yield evidence of malignancy. RT-ddPCR showed absence of molecular markers. OT grafts after 22-week xenotransplantation were absent of the markers by both – RT-ddPCR and IHC.

Risk factors/ determinants:
• Not all primary tumors exhibited both markers (n=4) by IHC.</td><td>Strengths:
• done all main three experimental methods for MID detection (IHC, RT-ddPCR, xenotransplantation);
• primary tumor control.

Limitations:
• all ovarian tissue samples investigated by immunohistochemistry but only 14 by RT-ddPCR.

Risk of bias
A. Selection bias:
high risk
Reason: low sample size

B. Attrition bias:
low risk
Reason: all patients gave an outcome: presence/absence of markers</td><td>C. Measurement bias:
moderate risk
Reason: not all patients had their ovarian tissue analyzed for markers by RT-ddPCR

D. Detection bias:
moderate risk
Reason: not all patients had their ovarian tissue analyzed for markers by RT-ddPCR

E. Confounding:
moderate risk
Reason: though all confounders (treatment before OTC, availability of original tumor/blood markers, survival status/disease recurrence) are accounted, not all of them are specified to which patient

F. Statistical analysis and reporting:
low risk
Reason: all data presented</td></tr>
</table>
